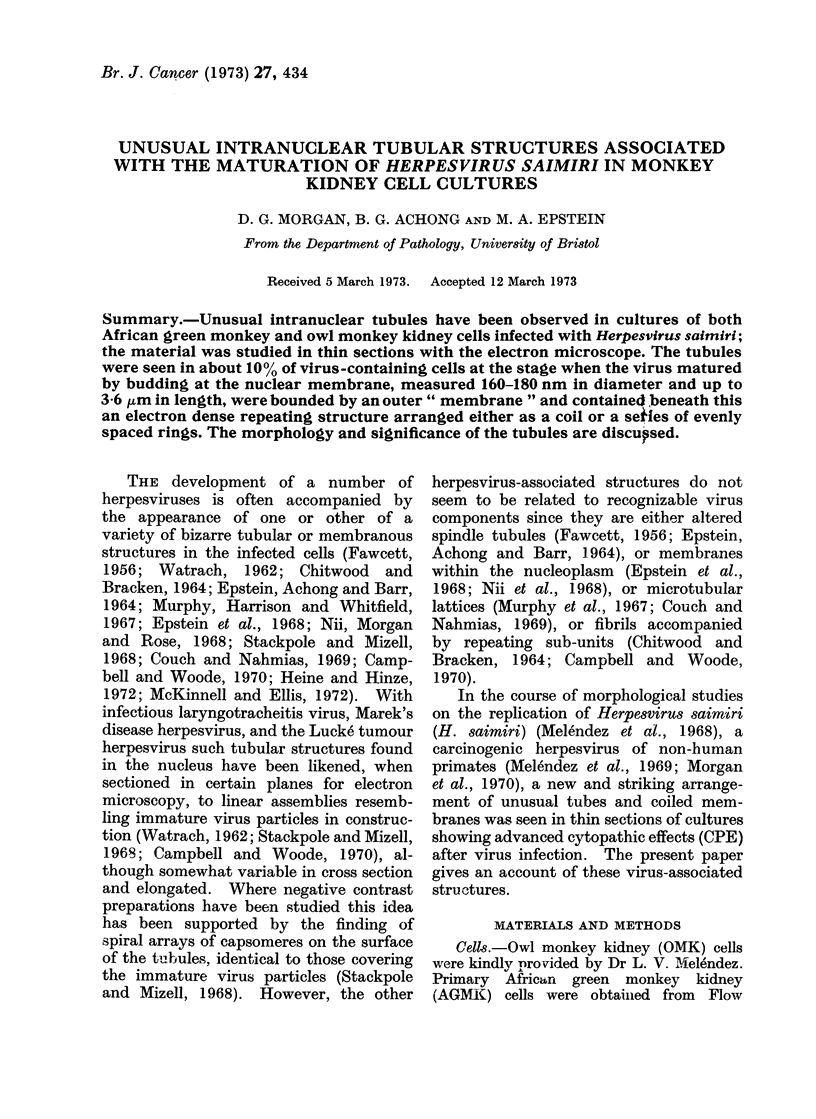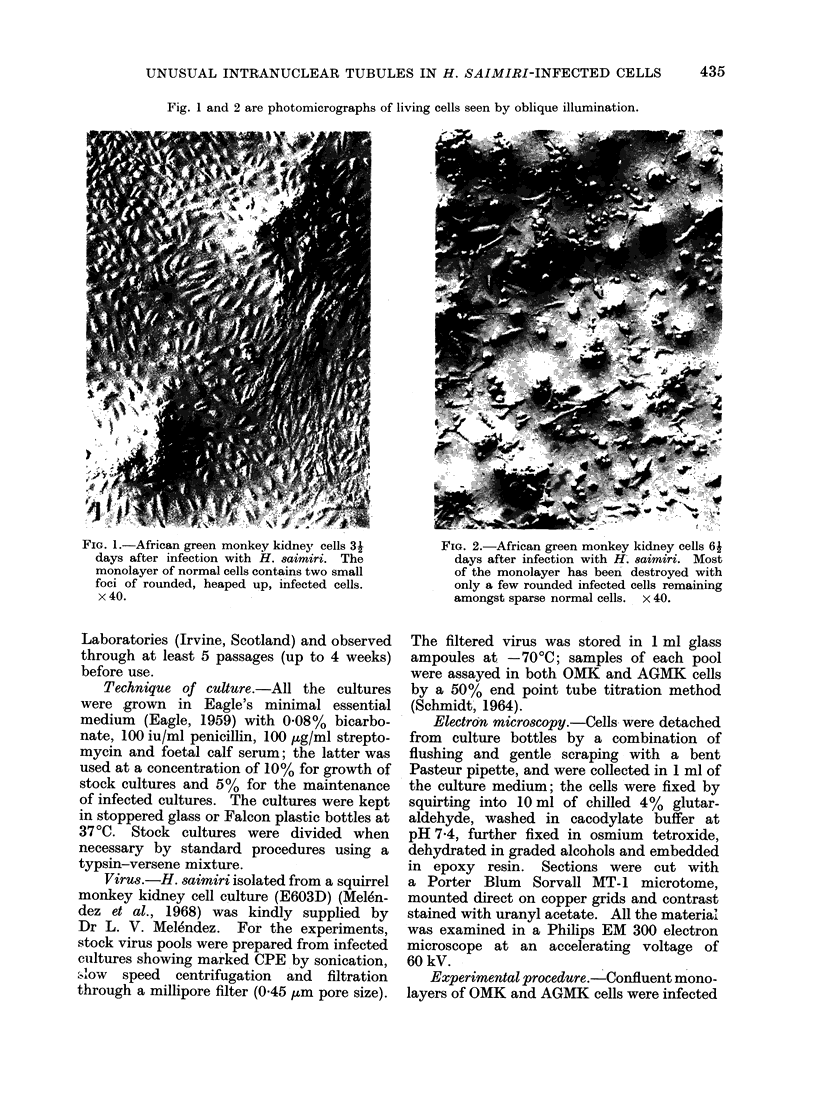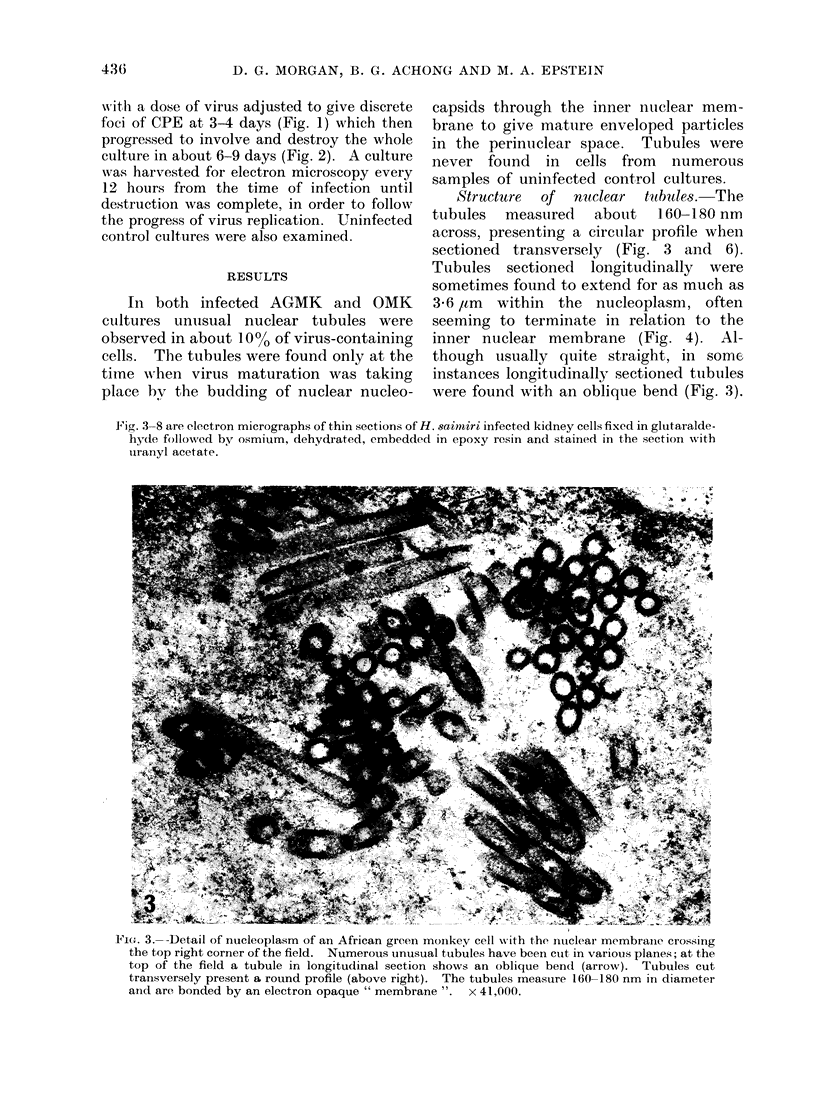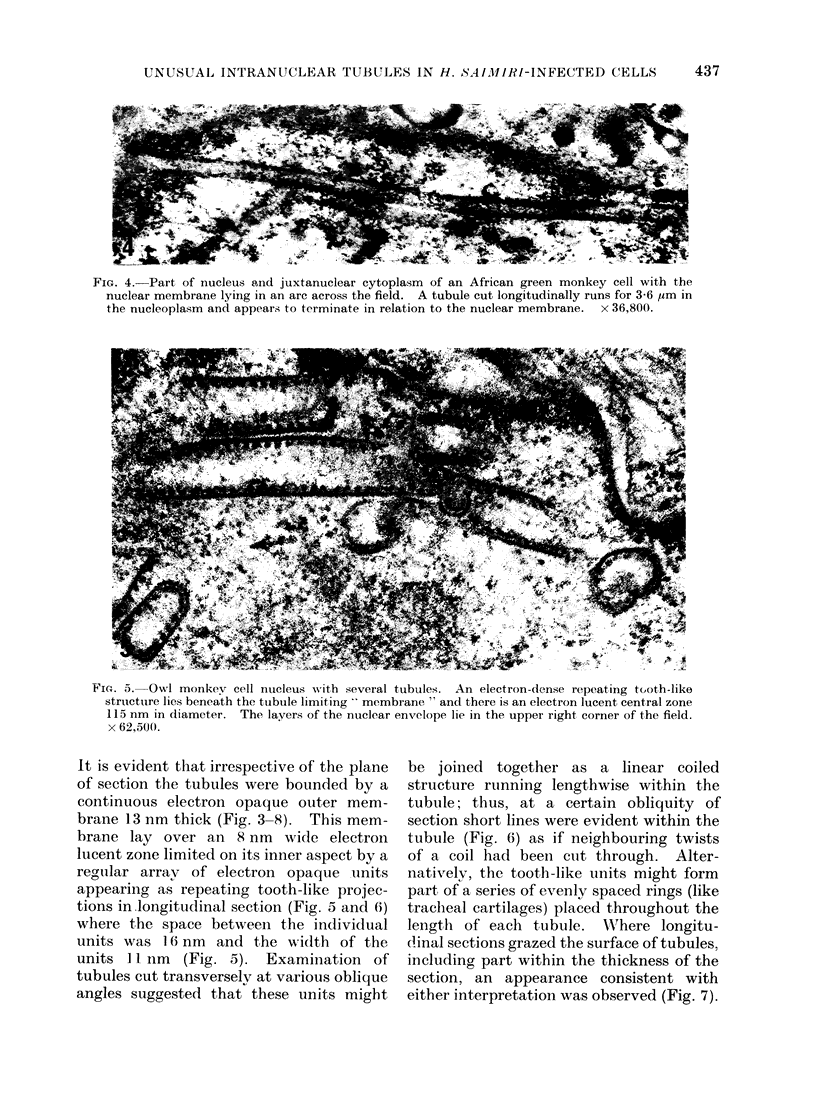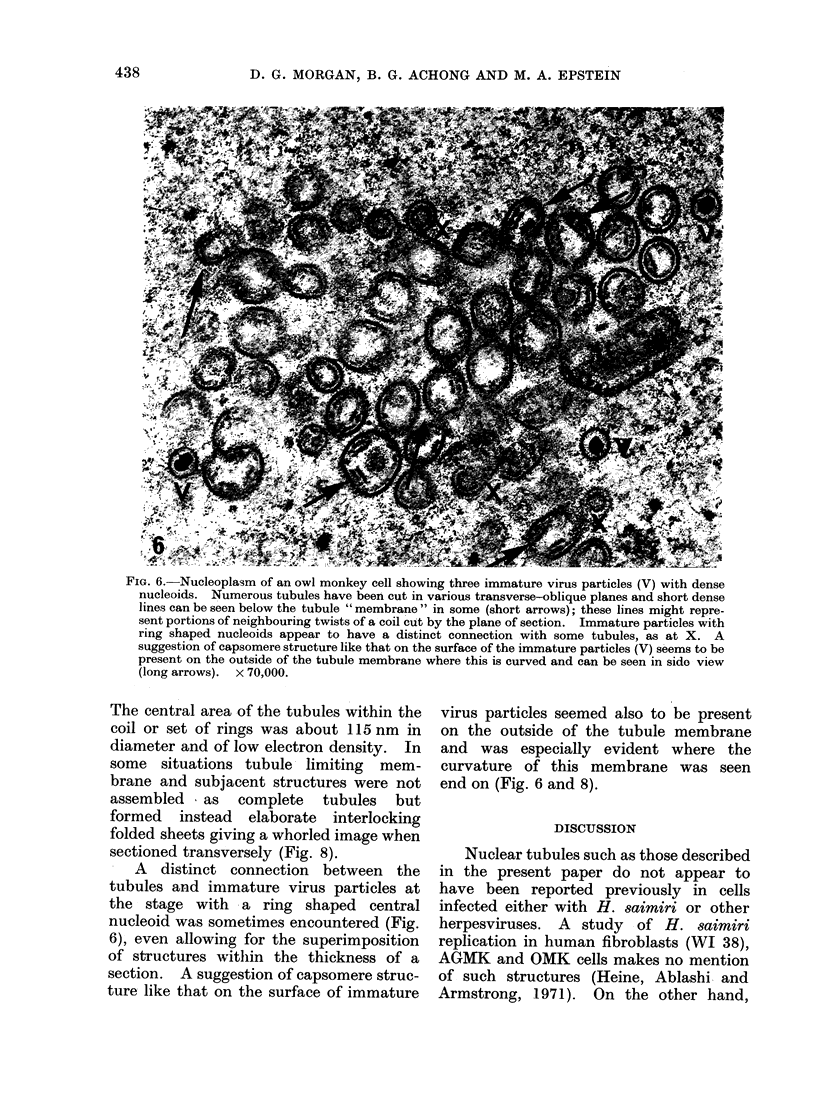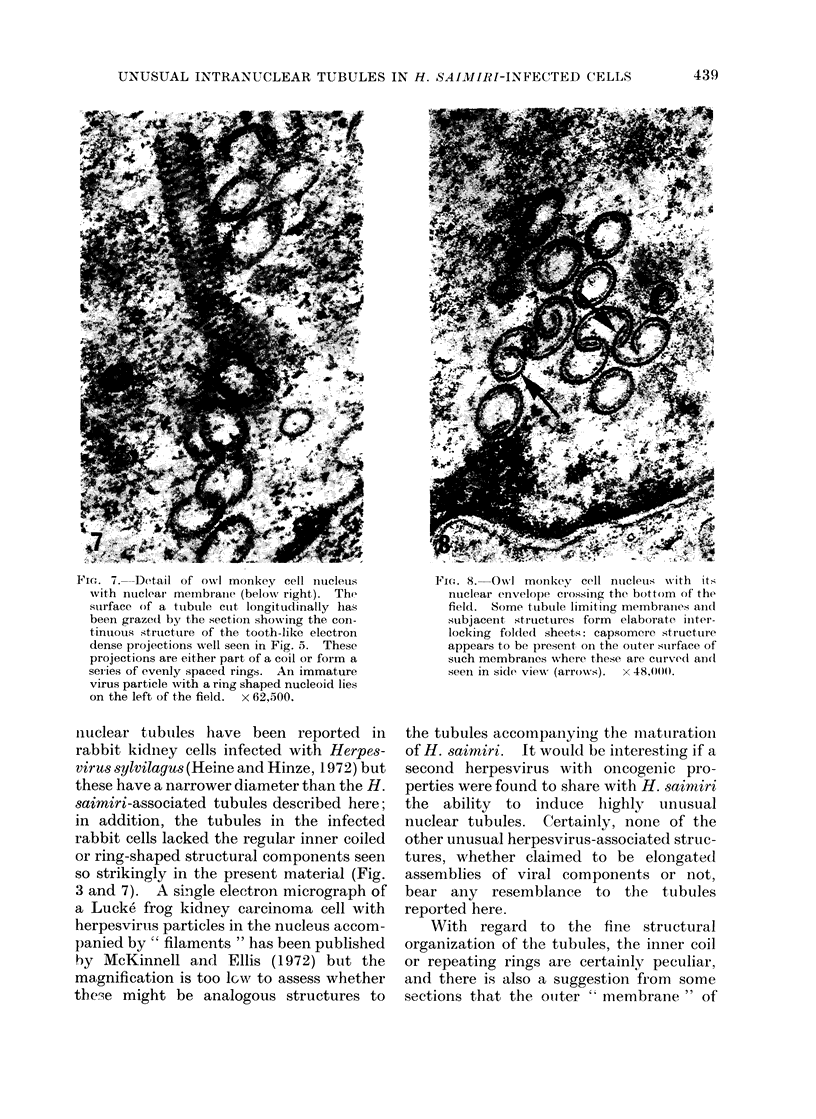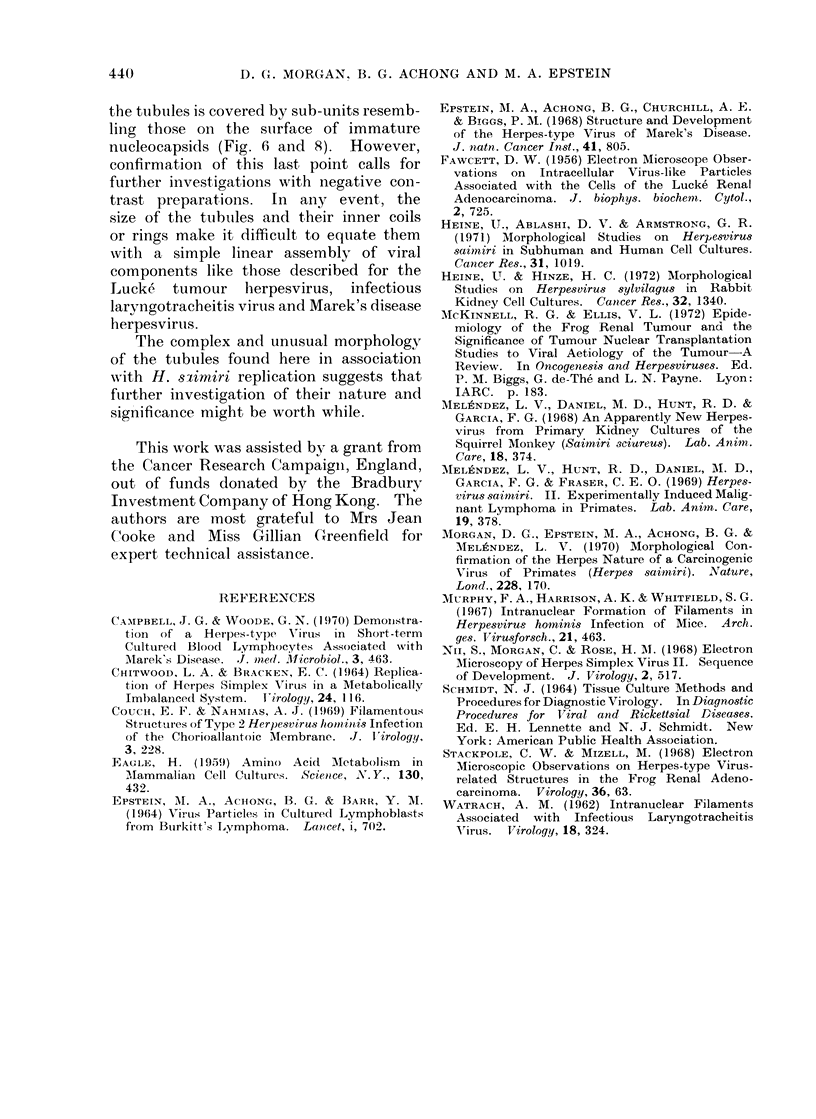# Unusual Intranuclear Tubular Structures Associated with the Maturation of Herpesvirus saimiri in Monkey Kidney Cell Cultures

**DOI:** 10.1038/bjc.1973.54

**Published:** 1973-06

**Authors:** D. G. Morgan, B. G. Achong, M. A. Epstein

## Abstract

**Images:**


*Br. J. Cancer* (1973) **27**, 434

### UNUSUAL INTRANUCLEAR TUBULAR STRUCTURES ASSOCIATED WITH THE MATURATION OF *HERPESVIRUS SAIMIRI* IN MONKEY KIDNEY CELL CULTURES

D. G. MORGAN, B. G. ACHONG AND M. A. EPSTEIN

*From the Department of Pathology, University of Bristol*

Received 5 March 1973. Accepted 12 March 1973

**Summary.—Unusual intranuclear tubules have been observed in cultures of both African green monkey and owl monkey kidney cells infected with *Herpesvirus saimiri*; the material was studied in thin sections with the electron microscope. The tubules were seen in about 10% of virus-containing cells at the stage when the virus matured by budding at the nuclear membrane, measured 160–180 nm in diameter and up to 3·6 μm in length, were bounded by an outer " membrane " and contained beneath this an electron dense repeating structure arranged either as a coil or a series of evenly spaced rings. The morphology and significance of the tubules are discussed.**

THE development of a number of herpesviruses is often accompanied by the appearance of one or other of a variety of bizarre tubular or membranous structures in the infected cells (Fawcett, 1956; Watrach, 1962; Chitwood and Bracken, 1964; Epstein, Achong and Barr, 1964; Murphy, Harrison and Whitfield, 1967; Epstein *et al.*, 1968; Nii, Morgan and Rose, 1968; Stackpole and Mizell, 1968; Couch and Nahmias, 1969; Campbell and Woode, 1970; Heine and Hinze, 1972; McKinnell and Ellis, 1972). With infectious laryngotracheitis virus, Marek's disease herpesvirus, and the Lucké tumour herpesvirus such tubular structures found in the nucleus have been likened, when sectioned in certain planes for electron microscopy, to linear assemblies resembling immature virus particles in construction (Watrach, 1962; Stackpole and Mizell, 1968; Campbell and Woode, 1970), although somewhat variable in cross section and elongated. Where negative contrast preparations have been studied this idea has been supported by the finding of spiral arrays of capsomeres on the surface of the tubules, identical to those covering the immature virus particles (Stackpole and Mizell, 1968). However, the other herpesvirus-associated structures do not seem to be related to recognizable virus components since they are either altered spindle tubules (Fawcett, 1956; Epstein, Achong and Barr, 1964), or membranes within the nucleoplasm (Epstein *et al.*, 1968; Nii *et al.*, 1968), or microtubular lattices (Murphy *et al.*, 1967; Couch and Nahmias, 1969), or fibrils accompanied by repeating sub-units (Chitwood and Bracken, 1964; Campbell and Woode, 1970).

In the course of morphological studies on the replication of *Herpesvirus saimiri* (*H. saimiri*) (Meléndez *et al.*, 1968), a carcinogenic herpesvirus of non-human primates (Meléndez *et al.*, 1969; Morgan *et al.*, 1970), a new and striking arrangement of unusual tubes and coiled membranes was seen in thin sections of cultures showing advanced cytopathic effects (CPE) after virus infection. The present paper gives an account of these virus-associated structures.

### MATERIALS AND METHODS

*Cells.*—Owl monkey kidney (OMK) cells were kindly provided by Dr L. V. Meléndez. Primary African green monkey kidney (AGMK) cells were obtained from Flow

Fig. 1 and 2 are photomicrographs of living cells seen by oblique illumination.

FIG. 1.—African green monkey kidney cells 3½ days after infection with *H. saimiri*. The monolayer of normal cells contains two small foci of rounded, heaped up, infected cells. ×40.

FIG. 2.—African green monkey kidney cells 6½ days after infection with *H. saimiri*. Most of the monolayer has been destroyed with only a few rounded infected cells remaining amongst sparse normal cells. ×40.

Laboratories (Irvine, Scotland) and observed through at least 5 passages (up to 4 weeks) before use.

*Technique of culture.*—All the cultures were grown in Eagle's minimal essential medium (Eagle, 1959) with 0·08% bicarbonate, 100 iu/ml penicillin, 100 μg/ml streptomycin and foetal calf serum; the latter was used at a concentration of 10% for growth of stock cultures and 5% for the maintenance of infected cultures. The cultures were kept in stoppered glass or Falcon plastic bottles at 37 °C. Stock cultures were divided when necessary by standard procedures using a typsin–versene mixture.

*Virus.*—*H. saimiri* isolated from a squirrel monkey kidney cell culture (E603D) (Meléndez *et al.*, 1968) was kindly supplied by Dr L. V. Meléndez. For the experiments, stock virus pools were prepared from infected cultures showing marked CPE by sonication, slow speed centrifugation and filtration through a millipore filter (0·45 μm pore size). The filtered virus was stored in 1 ml glass ampoules at −70 °C; samples of each pool were assayed in both OMK and AGMK cells by a 50% end point tube titration method (Schmidt, 1964).

*Electron microscopy.*—Cells were detached from culture bottles by a combination of flushing and gentle scraping with a bent Pasteur pipette, and were collected in 1 ml of the culture medium; the cells were fixed by squirting into 10 ml of chilled 4% glutaraldehyde, washed in cacodylate buffer at pH 7·4, further fixed in osmium tetroxide, dehydrated in graded alcohols and embedded in epoxy resin. Sections were cut with a Porter Blum Sorvall MT-1 microtome, mounted direct on copper grids and contrast stained with uranyl acetate. All the material was examined in a Philips EM 300 electron microscope at an accelerating voltage of 60 kV.

*Experimental procedure.*—Confluent monolayers of OMK and AGMK cells were infected with a dose of virus adjusted to give discrete foci of CPE at 3–4 days (Fig. 1) which then progressed to involve and destroy the whole culture in about 6–9 days (Fig. 2). A culture was harvested for electron microscopy every 12 hours from the time of infection until destruction was complete, in order to follow the progress of virus replication. Uninfected control cultures were also examined.

### RESULTS

In both infected AGMK and OMK cultures unusual nuclear tubules were observed in about 10% of virus-containing cells. The tubules were found only at the time when virus maturation was taking place by the budding of nuclear nucleocapsids through the inner nuclear membrane to give mature enveloped particles in the perinuclear space. Tubules were never found in cells from numerous samples of uninfected control cultures.

*Structure of nuclear tubules.*—The tubules measured about 160–180 nm across, presenting a circular profile when sectioned transversely (Fig. 3 and 6). Tubules sectioned longitudinally were sometimes found to extend for as much as 3·6 $\mu$m within the nucleoplasm, often seeming to terminate in relation to the inner nuclear membrane (Fig. 4). Although usually quite straight, in some instances longitudinally sectioned tubules were found with an oblique bend (Fig. 3).

Fig. 3–8 are electron micrographs of thin sections of *H. saimiri* infected kidney cells fixed in glutaraldehyde followed by osmium, dehydrated, embedded in epoxy resin and stained in the section with uranyl acetate.

FIG. 3.—Detail of nucleoplasm of an African green monkey cell with the nuclear membrane crossing the top right corner of the field. Numerous unusual tubules have been cut in various planes; at the top of the field a tubule in longitudinal section shows an oblique bend (arrow). Tubules cut transversely present a round profile (above right). The tubules measure 160–180 nm in diameter and are bonded by an electron opaque " membrane ". × 41,000.

FIG. 4.—Part of nucleus and juxtanuclear cytoplasm of an African green monkey cell with the nuclear membrane lying in an arc across the field. A tubule cut longitudinally runs for 3·6 μm in the nucleoplasm and appears to terminate in relation to the nuclear membrane. ×36,800.

FIG. 5.—Owl monkey cell nucleus with several tubules. An electron-dense repeating tooth-like structure lies beneath the tubule limiting " membrane " and there is an electron lucent central zone 115 nm in diameter. The layers of the nuclear envelope lie in the upper right corner of the field. ×62,500.

It is evident that irrespective of the plane of section the tubules were bounded by a continuous electron opaque outer membrane 13 nm thick (Fig. 3–8). This membrane lay over an 8 nm wide electron lucent zone limited on its inner aspect by a regular array of electron opaque units appearing as repeating tooth-like projections in longitudinal section (Fig. 5 and 6) where the space between the individual units was 16 nm and the width of the units 11 nm (Fig. 5). Examination of tubules cut transversely at various oblique angles suggested that these units might be joined together as a linear coiled structure running lengthwise within the tubule; thus, at a certain obliquity of section short lines were evident within the tubule (Fig. 6) as if neighbouring twists of a coil had been cut through. Alternatively, the tooth-like units might form part of a series of evenly spaced rings (like tracheal cartilages) placed throughout the length of each tubule. Where longitudinal sections grazed the surface of tubules, including part within the thickness of the section, an appearance consistent with either interpretation was observed (Fig. 7).

FIG. 6.—Nucleoplasm of an owl monkey cell showing three immature virus particles (V) with dense nucleoids. Numerous tubules have been cut in various transverse–oblique planes and short dense lines can be seen below the tubule "membrane" in some (short arrows); these lines might represent portions of neighbouring twists of a coil cut by the plane of section. Immature particles with ring shaped nucleoids appear to have a distinct connection with some tubules, as at X. A suggestion of capsomere structure like that on the surface of the immature particles (V) seems to be present on the outside of the tubule membrane where this is curved and can be seen in side view (long arrows). × 70,000.

The central area of the tubules within the coil or set of rings was about 115 nm in diameter and of low electron density. In some situations tubule limiting membrane and subjacent structures were not assembled as complete tubules but formed instead elaborate interlocking folded sheets giving a whorled image when sectioned transversely (Fig. 8).

A distinct connection between the tubules and immature virus particles at the stage with a ring shaped central nucleoid was sometimes encountered (Fig. 6), even allowing for the superimposition of structures within the thickness of a section. A suggestion of capsomere structure like that on the surface of immature virus particles seemed also to be present on the outside of the tubule membrane and was especially evident where the curvature of this membrane was seen end on (Fig. 6 and 8).

### DISCUSSION

Nuclear tubules such as those described in the present paper do not appear to have been reported previously in cells infected either with *H. saimiri* or other herpesviruses. A study of *H. saimiri* replication in human fibroblasts (WI 38), AGMK and OMK cells makes no mention of such structures (Heine, Ablashi and Armstrong, 1971). On the other hand,

FIG. 7.—Detail of owl monkey cell nucleus with nuclear membrane (below right). The surface of a tubule cut longitudinally has been grazed by the section showing the continuous structure of the tooth-like electron dense projections well seen in Fig. 5. These projections are either part of a coil or form a series of evenly spaced rings. An immature virus particle with a ring shaped nucleoid lies on the left of the field. ×62,500.

FIG. 8.—Owl monkey cell nucleus with its nuclear envelope crossing the bottom of the field. Some tubule limiting membranes and subjacent structures form elaborate interlocking folded sheets: capsomere structure appears to be present on the outer surface of such membranes where these are curved and seen in side view (arrows). ×48,000.

nuclear tubules have been reported in rabbit kidney cells infected with *Herpesvirus sylvilagus* (Heine and Hinze, 1972) but these have a narrower diameter than the *H. saimiri*-associated tubules described here; in addition, the tubules in the infected rabbit cells lacked the regular inner coiled or ring-shaped structural components seen so strikingly in the present material (Fig. 3 and 7). A single electron micrograph of a Lucké frog kidney carcinoma cell with herpesvirus particles in the nucleus accompanied by " filaments " has been published by McKinnell and Ellis (1972) but the magnification is too low to assess whether these might be analogous structures to the tubules accompanying the maturation of *H. saimiri*. It would be interesting if a second herpesvirus with oncogenic properties were found to share with *H. saimiri* the ability to induce highly unusual nuclear tubules. Certainly, none of the other unusual herpesvirus-associated structures, whether claimed to be elongated assemblies of viral components or not, bear any resemblance to the tubules reported here.

With regard to the fine structural organization of the tubules, the inner coil or repeating rings are certainly peculiar, and there is also a suggestion from some sections that the outer " membrane " of the tubules is covered by sub-units resembling those on the surface of immature nucleocapsids (Fig. 6 and 8). However, confirmation of this last point calls for further investigations with negative contrast preparations. In any event, the size of the tubules and their inner coils or rings make it difficult to equate them with a simple linear assembly of viral components like those described for the Lucké tumour herpesvirus, infectious laryngotracheitis virus and Marek's disease herpesvirus.

The complex and unusual morphology of the tubules found here in association with *H. saimiri* replication suggests that further investigation of their nature and significance might be worth while.

This work was assisted by a grant from the Cancer Research Campaign, England, out of funds donated by the Bradbury Investment Company of Hong Kong. The authors are most grateful to Mrs Jean Cooke and Miss Gillian Greenfield for expert technical assistance.

### REFERENCES

Campbell, J. G. & Woode, G. N. (1970) Demonstration of a Herpes-type Virus in Short-term Cultured Blood Lymphocytes Associated with Marek's Disease. *J. med. Microbiol.*, **3**, 463.

Chitwood, L. A. & Bracken, E. C. (1964) Replication of Herpes Simplex Virus in a Metabolically Imbalanced System. *Virology*, **24**, 116.

Couch, E. F. & Nahmias, A. J. (1969) Filamentous Structures of Type 2 *Herpesvirus hominis* Infection of the Chorioallantoic Membrane. *J. Virology*, **3**, 228.

Eagle, H. (1959) Amino Acid Metabolism in Mammalian Cell Cultures. *Science, N.Y.*, **130**, 432.

Epstein, M. A., Achong, B. G. & Barr, Y. M. (1964) Virus Particles in Cultured Lymphoblasts from Burkitt's Lymphoma. *Lancet*, i, 702.

Epstein, M. A., Achong, B. G., Churchill, A. E. & Biggs, P. M. (1968) Structure and Development of the Herpes-type Virus of Marek's Disease. *J. natn. Cancer Inst.*, **41**, 805.

Fawcett, D. W. (1956) Electron Microscope Observations on Intracellular Virus-like Particles Associated with the Cells of the Lucké Renal Adenocarcinoma. *J. biophys. biochem. Cytol.*, **2**, 725.

Heine, U., Ablashi, D. V. & Armstrong, G. R. (1971) Morphological Studies on *Herpesvirus saimiri* in Subhuman and Human Cell Cultures. *Cancer Res.*, **31**, 1019.

Heine, U. & Hinze, H. C. (1972) Morphological Studies on *Herpesvirus sylvilagus* in Rabbit Kidney Cell Cultures. *Cancer Res.*, **32**, 1340.

McKinnell, R. G. & Ellis, V. L. (1972) Epidemiology of the Frog Renal Tumour and the Significance of Tumour Nuclear Transplantation Studies to Viral Aetiology of the Tumour—A Review. In *Oncogenesis and Herpesviruses*. Ed. P. M. Biggs, G. de-Thé and L. N. Payne. Lyon: IARC. p. 183.

Meléndez, L. V., Daniel, M. D., Hunt, R. D. & Garcia, F. G. (1968) An Apparently New Herpesvirus from Primary Kidney Cultures of the Squirrel Monkey (*Saimiri sciureus*). *Lab. Anim. Care*, **18**, 374.

Meléndez, L. V., Hunt, R. D., Daniel, M. D., Garcia, F. G. & Fraser, C. E. O. (1969) *Herpesvirus saimiri*. II. Experimentally Induced Malignant Lymphoma in Primates. *Lab. Anim. Care*, **19**, 378.

Morgan, D. G., Epstein, M. A., Achong, B. G. & Meléndez, L. V. (1970) Morphological Confirmation of the Herpes Nature of a Carcinogenic Virus of Primates (*Herpes saimiri*). *Nature, Lond.*, **228**, 170.

Murphy, F. A., Harrison, A. K. & Whitfield, S. G. (1967) Intranuclear Formation of Filaments in *Herpesvirus hominis* Infection of Mice. *Arch. ges. Virusforsch.*, **21**, 463.

Nii, S., Morgan, C. & Rose, H. M. (1968) Electron Microscopy of Herpes Simplex Virus II. Sequence of Development. *J. Virology*, **2**, 517.

Schmidt, N. J. (1964) Tissue Culture Methods and Procedures for Diagnostic Virology. In *Diagnostic Procedures for Viral and Rickettsial Diseases*. Ed. E. H. Lennette and N. J. Schmidt. New York: American Public Health Association.

Stackpole, C. W. & Mizell, M. (1968) Electron Microscopic Observations on Herpes-type Virus-related Structures in the Frog Renal Adenocarcinoma. *Virology*, **36**, 63.

Watrach, A. M. (1962) Intranuclear Filaments Associated with Infectious Laryngotracheitis Virus. *Virology*, **18**, 324.